# Gene Therapy for β-Hemoglobinopathies: From Discovery to Clinical Trials

**DOI:** 10.3390/v15030713

**Published:** 2023-03-09

**Authors:** Eva Eugenie Rose Segura, Paul George Ayoub, Kevyn Lopez Hart, Donald Barry Kohn

**Affiliations:** 1 Molecular Biology Interdepartmental Doctoral Program, David Geffen School of Medicine, University of California, Los Angeles, CA 90095, USA; evarosesegura@ucla.edu; 2 Department of Molecular & Medical Pharmacology, David Geffen School of Medicine, University of California, Los Angeles, CA 90095, USA; 3 Department of Human Genetics, David Geffen School of Medicine, University of California, Los Angeles, CA 90095, USA; 4 Department of Microbiology, Immunology & Molecular Genetics, David Geffen School of Medicine, University of California, Los Angeles, CA 90095, USA; 5 Department of Pediatrics (Hematology/Oncology), David Geffen School of Medicine, University of California, Los Angeles, CA 90095, USA; 6 Eli and Edythe Broad Center for Stem Cell Research and Regenerative Medicine, University of California, Los Angeles, CA 90095, USA

**Keywords:** gene therapy, autologous hematopoietic stem-cell transplant, β-hemoglobinopathies, β-thalassemia, sickle cell disease, lentiviral vectors, clinical trials, β-globin genes

## Abstract

Investigations to understand the function and control of the globin genes have led to some of the most exciting molecular discoveries and biomedical breakthroughs of the 20th and 21st centuries. Extensive characterization of the globin gene locus, accompanied by pioneering work on the utilization of viruses as human gene delivery tools in human hematopoietic stem and progenitor cells (HPSCs), has led to transformative and successful therapies via autologous hematopoietic stem-cell transplant with gene therapy (HSCT-GT). Due to the advanced understanding of the β-globin gene cluster, the first diseases considered for autologous HSCT-GT were two prevalent β-hemoglobinopathies: sickle cell disease and β-thalassemia, both affecting functional β-globin chains and leading to substantial morbidity. Both conditions are suitable for allogeneic HSCT; however, this therapy comes with serious risks and is most effective using an HLA-matched family donor (which is not available for most patients) to obtain optimal therapeutic and safe benefits. Transplants from unrelated or haplo-identical donors carry higher risks, although they are progressively improving. Conversely, HSCT-GT utilizes the patient’s own HSPCs, broadening access to more patients. Several gene therapy clinical trials have been reported to have achieved significant disease improvement, and more are underway. Based on the safety and the therapeutic success of autologous HSCT-GT, the U.S. Food and Drug Administration (FDA) in 2022 approved an HSCT-GT for β-thalassemia (Zynteglo™). This review illuminates the β-globin gene research journey, adversities faced, and achievements reached; it highlights important molecular and genetic findings of the β-globin locus, describes the predominant globin vectors, and concludes by describing promising results from clinical trials for both sickle cell disease and β-thalassemia.

## 1. Introduction

In the 20th century, the scientific community took a keen interest in globin genes. Globin research spurred scientists from various fields of biology, from molecular to structural, to extensively deepen our scientific knowledge. Such convergence of diverse scientific studies resulted in major discoveries: β-globin transcriptional regulatory elements were the first identified distal regulatory elements to control a gene cluster; sickle cell disease was the first condition to be elucidated at the molecular level; the β-globin promoter was one of the first eukaryotic promoters to be extensively characterized; and hemoglobin was the first complex protein to be resolved in three dimensions [1,2,3,4]. Notably, globin research enabled for the first time the utilization of viruses as biological tools; a revolutionary application that made unfathomable therapeutic goals into an imminent reality. While research on globin genes was hindered by various scientific challenges and a concurrently evolving technology, it ultimately achieved one of the first clinical successes through autologous hematopoietic stem-cell transplant gene therapy (HSCT-GT).

**Figure 1:**
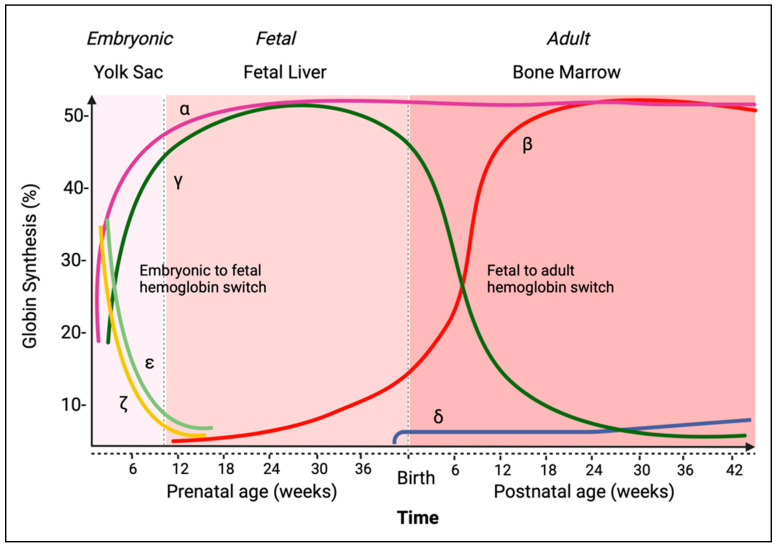
Temporal expression of globin chains. The assembly of two α-like globin chains with its two appropriate β-like globin chains produce functional tetrameric hemoglobin. Globin expression begins in the first weeks of development in the yolk sac, with the production of the primary embryonic hemoglobin, Hemoglobin Gower (HbE-Gower-1) consisting of the embryonic beta-like globin chain or epsilon-globin (ε), and the embryonic alpha-like globin chain, zeta-globin (ζ). During fetal development, starting at week 10, the primary fetal hemoglobin produced is fetal hemoglobin (HbF) consisting of two fetal beta-like globin chains, gamma-globin (γ), along with the alpha-globin chains (α), whose expression will remain active postnatally. The fetal γ-globin chain, however, decreases rapidly postnatally and is replaced by the adult beta-globin chain (β), which together with the α-globin chain produces the adult hemoglobin (HbA). A secondary adult β-like globin gene, delta-globin (δ) is also expressed postnatally but remains at a low concentration throughout life and assembles with the α-globin chain to form hemoglobin (HbA2).

The human β-globin locus, located on the short arm of chromosome 11, encodes for five globin genes arranged in order of their developmental expression: the embryonic epsilon, ε, gene (*HBE1*) expressed in the yolk sac; the fetal gamma, Gγ and Aγ genes (*HBG2*, *HBG1*), mostly expressed during hematopoiesis in the fetal liver; and finally, the adult delta, δ, and beta, β, genes (*HBD*, *HBB*), expressed in erythrocytes in the bone marrow and blood after birth. Conjunctly, the human α-globin locus, located at the end of chromosome 16, encodes for three genes, also arranged in order of their developmental expression: the embryonic zeta, ζ, *(HBZ*), and two alpha, α_2_ and α_1_ (*HBA2* and *HBA1*), both expressed at the fetal and adult stages. The primary hemoglobin produced during development is fetal hemoglobin (HbF), constituted of two α-globin and two γ-globin chains. After birth, adult hemoglobin (HbA) is the most predominant globin, constituted of two α-globin and two β-globin chains (Figure 1, modified from Hemoglobin Structure, Human Hemoglobin. 1977) [5]. Research on the developmental complexity and tissue restriction of β-globin genes not only advanced the field of molecular biology but also allowed the development of new molecular tools and technologies. Starting with the development of in vivo experiments: the rabbit adult β-globin gene was utilized to generate the first transgenic mouse line via embryonic microinjection of DNA sequences [6]. This exploit further facilitated and heightened interest in β-globin research which became a scientific focus for the following decades, including a major focus at the recurring annual meeting known as the Hemoglobin Switching Meetings.

Because of the low through-put of the early microinjection technique, methods for β-globin gene incorporation into cells were quickly replaced by less harmful and more natural gene delivery systems which exploited viruses for gene delivery. The successful integration of foreign genetic information into hematopoietic stem and progenitor cells (HSPCs) inspired scientists to develop viral gene therapies for lympho-hematopoietic disorders via viral vectors. Owing to the extensive characterization of β-globin genes, the first diseases considered for gene therapy research were disorders caused by mutation(s) in the β-globin gene, such as sickle cell disease and β-thalassemia, both resulting in dysfunctional or reduced amounts of the β-globin subunit of hemoglobin in red blood cells (RBCs), respectively. Additionally, interest and extensive research in the fetal globin genes emerged with the observations that sickle cell disease patients with abnormally elevated γ-globin levels experience a less severe course of the disease [7,8,9].

This review will summarize β-globin research enabled by continuously evolving viral biotechnologies, highlighting influential findings and setbacks, as well as emerging technological advancements that fostered both γ- and β-globin vector research and, eventually, successful gene therapies for sickle cell disease and β-thalassemia.

## 2. The β-Globin Locus Control Region (LCR)

**Figure 2:**
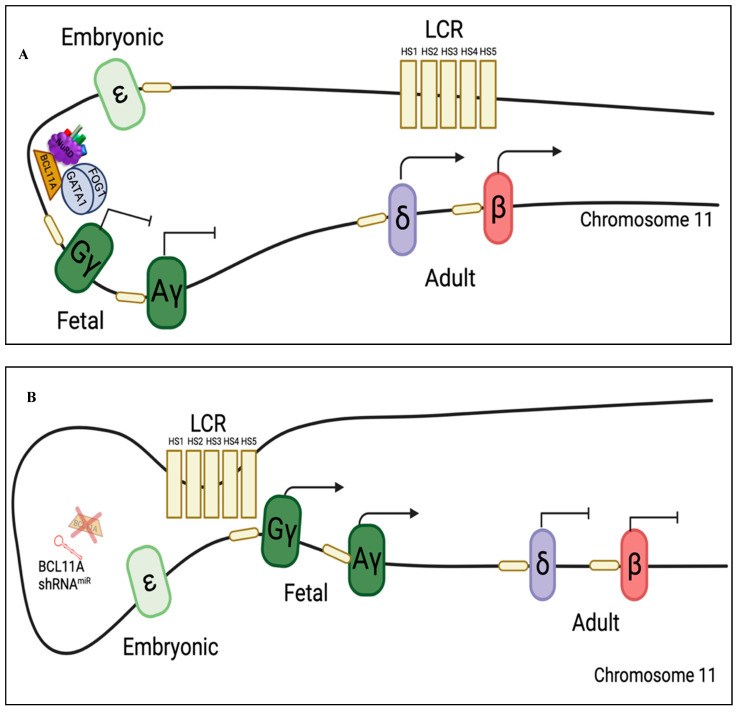
Mechanism of HbF regulation. (**A**) Endogenous HbF silencing. The human β-globin locus is comprised of the Locus Control Region (LCR) which regulates and enhances the expression of each of the globin genes through chromatin looping. When hemoglobin switching occurs from HbF to HbA expression, the nucleosome remodeling and deacetylase (NuRD) complex, FOG1, GATA1, and B Cell Lymphoma 11A (BCL11A) bind to the fetal globin promoters, leading to the repression of fetal globin expression and the activation of the adult globin genes. (**B**) Mechanism of BCL11A shRNA^miR^ silencing. The developed BCL11A shRNA^miR^ binds to BCL11A mRNA and targets it for breakdown in the cell, subsequently depleting BCL11A protein. Such a process prevents the BCL11A protein and protein complex binding to the fetal globin promoter binding site, thereby inducing γ-globin expression.

In the mid-1980s, shortly after the generation of β-globin transgenic mouse models, researchers uncovered essential information on the β-globin gene locus. Promoters were identified at the 5′ end of each of the five genes, establishing that expression of each of the genes was driven individually [10]. Large and distal erythroid-specific DNase I hypersensitive regions flanking the β-globin cluster were identified in both human erythroleukemic cells and K562 cells, a human immortalized chronic myelogenous leukemia cell line that can be induced to erythroid differentiation, expressing mainly HbF [11]. The 5′ hypersensitive region, ranging from 6 kb to 40 kb upstream of the ε-globin gene, was further divided into five distinctive DNase I hypersensitive sites (HS): HS1, HS2, HS3, HS4, and HS5 (Figure 2A).

Because in vitro and in vivo experiments would not yield consistent nor proper β-globin gene expression, the discovery of the large HS region, made by London’s group, sparked interest and encouraged scientists to investigate its role in globin expression [11]. By 1987, the Groudine laboratory had identified a ‘minilocus’ possessing erythroid-specific expression at a level directly related to its copy number while being independent of its position of integration [12]. This ‘minilocus’, later referred to as the Locus Control Region (LCR), reinforced the possibility of the existence of distal *cis*-regulatory elements.

By the 2000s, numerous transcription factor binding sites and important erythroid regulatory elements associated with the β-globin locus were identified. Erythroid lineage-restricted Kruppel-like transcription factor (KLF1), a member of the Kruppel-like factor (EKLF) family, was one of the first elements shown to have an important role in γ- to β-globin genes switching [13,14]. This conclusion was supported by its high affinity to the β-globin promoter compared to the γ-globin counterpart, and its high concentration in adult erythroid tissues [13,15]. At the same time, EKLF-DNA binding site mutations were identified in β-thalassemia patients, further supporting the importance and clinical relevance of KLF1 for regulating β-globin expression [16,17]. Additionally, GATA-1, a zinc finger transcription and master regulator with broad effects on numerous genes involved at the early stages of erythroid differentiation, was first identified by its ability to bind to regulatory DNA sequences of the globin genes [18,19]. Further analysis determined GATA-1 to be an erythroid transcriptional activator, based on its roles in histone acetylation on the β-LCR, and, along with NF-E2, on chromatin looping—both of which allowed for proper γ-globin expression [20]. TAL1, a binding partner of GATA-1, is another critical factor in erythropoiesis whose expression was shown to be essential in embryonic stem cells for globin gene expression [21,22,23,24]. In addition to GATA-1, TAL1 was demonstrated to play an integral part in chromatin looping for γ-globin expression [25]. Furthermore, these three factors, along with LMO2, were shown to form a complex with Ldb1 that facilitated and stabilized long-distance interaction between the β-LCR enhancer elements and the erythroid promoters [26,27]. Overall, KLF1, GATA-1 and TAL1 were discovered early on in globin research and were identified as erythroid-restricted transcription factors whose activities were demonstrated as necessary for primitive and definitive erythropoiesis. Additional studies also determined their functions are essential both for β-LCR loop formation and expression of β-, γ- and α-globin genes. Initial findings of the regulation that governs globin genes readily exposed the complexity of comprehending and modulating appropriate hemoglobin production in a tissue- and cell-restricted manner.

Elucidation of the networks regulating the globin genes has long been of interest to the scientific community. Specifically, the drive to understand the underlying mechanisms of γ- and β-globin gene switching was heightened after the discovery that patients with abnormally high HbF experience less severe clinical manifestations from sickle cell disease and β-thalassemia [8,9,28]. With such efforts, many of the erythroid-specific regulatory elements strongly involved in γ- and β-globin gene regulation, activation, or repression were investigated and, for some, utilized as putative therapeutic targets (Described in Section 5). Numerous factors have been revealed and thoroughly examined with advancing biological technologies. One of the more dominant elements, now well-understood in γ-globin gene expression regulation, is the zinc-finger protein B Cell Lymphoma 11A (BCL11A), a potent γ-globin repressor [29,30,31]. Studies suggest that BCL11A interacts with GATA1, FOG1, SRY-box 6 (SOX6), the nucleosome remodeling and deacetylase complex (NuRD), and DNA methyltransferase 1 (DNMT1) to silence γ-globin expression via binding on its distal and proximal promoters [30,32,33,34]. ZNF410, another major repressor, blocks γ-globin expression by increasing the transcription of *CHD4*, from which the NuRD complex is encoded [35]. Studies have also shown that MYB binds to the β-globin activator KLF1 promoter, transactivates KLF1 expression, and in turn activates the γ-globin repressor GATA-1. Thus, MYB is now characterized as a potent γ-globin repressor [36]. Further supporting their involvement in globin switching, BCL11A, MYB, and HBB genetic variants have been measured to account for 10–20% of HbF variation [37,38]. Moreover, a study showed that hydroxyurea, a U.S. Food and Drug Administration (FDA)-approved pharmacologic treatment known for improving sickle cell disease and β-thalassemia symptoms and crises, induces HbF [39,40]. While the mechanism(s) of hydroxyurea on HbF induction are not totally understood, some studies have demonstrated its involvement in the reduction of BCL11A, MYB and KLF1, further highlighting the importance of these factors as therapeutic targets for β-hemoglobinopathies [41]. Overall, efforts from the last decades have shed enough light on the mechanisms that orchestrate hemoglobin switching for developing gene therapy approaches targeting such discovered regulators (e.g., BCL11A, ZNF410) for sickle cell disease and β-thalassemia. Nonetheless, more research is still needed to completely unravel this dynamic, interconnected network.

## 3. β-Globin Retroviral Vectors

In 1984, the first utilization of retroviruses to successfully and non-detrimentally incorporate a foreign gene into murine HSPCs was demonstrated [42]. This novel and suitable gene delivery tool presented the possibility to treat human hematopoietic disorders. Viral gene delivery techniques offered an alternative and natural biological process that circumvented the limitations of the microinjection technique while still retaining chromosomal integration of the transgene. Viral vectors are engineered virion particles in which the genetic elements needed for pathogenicity and viral replication are removed and replaced by the transgene of interest. Unlike microinjection, viral-mediated gene transfer is not restricted to one-cell embryos, but can, in theory, transduce most cells throughout prenatal and postnatal development. The effective gene transfer by retroviral vectors into enough repopulating HSPCs was ideal for clinical application to autologous HSPCs gene therapy.

By 1986, the first transgenic mice carrying β-globin genes introduced by retroviral vectors were produced [43]. Soriano et al. showed that β-globin expression driven by its own internal promoter was responsive to trans-acting developmental signals. They also noted that higher viral titer could be obtained only when the genomic sequences of the β-globin genes were in reverse orientation to the transcriptional direction of the provirus, avoiding intron splicing before integration into the genome. Rapidly confirmed by different laboratories, these two components (incorporation of β-globin regulatory elements and introns, and placement of the expression cassette in reverse orientation) have remained integral parts of β-globin vectors. Further, sequences present within intron 2 were identified for their deleterious to vector titer and are generally deleted at variable lengths in most current β-globin vectors.

In 1988, the first successful retroviral gene transfer of the β-globin gene into HSPCs transplanted to lethally irradiated mice enabled the reconstitution of bone marrow cells with human β-globin generation [44]. Such results presented the possibility to obtain high cell-specificity and long-term β-globin expression. However, the β-globin expression levels were minimal, an outcome often observed by different groups.

Numerous investigations have been conducted to incorporate regions of the LCR within retroviral vectors to confer erythroid-specific and position-independent expression. Smaller regions of the LCR are necessary to not only confer temporal and elevated β-globin expression but also to produce vector genomes within the normal size range of retroviral genomic lengths (~8–9 kb). Although extensive studies conducted from the mid-1980s to mid-1990s characterized the functions and boundaries of the LCR HS regions on β-globin gene expression, data of retroviral vectors incorporating various LCR regions were often ambiguous and inconsistent. The retroviral vectors yielded either low titers, low gene transfer or unstable vector genomes, inadequate β-globin gene expression, position-dependent expression, or a combination of these ineffective outcomes.

In 1995, Sadelain and team identified “core” regulatory element regions (HS2, HS3 and HS4) which successfully generated high titer retroviral vectors and expressed elevated β-globin mRNA in erythroid differentiated murine erythroleukemia cells (MEL) [45]. For the first time, a viral vector generated an efficient transduction and β-globin gene transfer, while retaining erythroid-specific expression at an elevated level. The major drawback of this study, however, was the high variability of the results. By reducing the length of the HSs to their core regions, position-independent expression was lost, and clone-to-clone variation in β-globin expression levels in MEL cells was present.

Overall, retroviral-mediated transfer in mouse HSPCs enabled erythroid-specific β-globin expression with the inclusion of regions from HS2, HS3 and HS4. Vectors containing large LCR fragments yielded erythroid β-globin expression; however, their large size exceeded retroviruses’ packaging capacity and caused low viral titers and inefficient gene transfer. The inclusion of a minimized LCR, on the other hand, yielded elevated expression but was subjected to a chromosomal position effect.

## 4. Lentiviral Vectors

In the mid-1990s, investigators at the Salk Institute developed efficient gene delivery vectors derived from the lentivirus Human Immunodeficiency Virus (HIV-1), a member of the Retroviridae family. These lentiviral vectors (LVs) offered several distinct advantages over the γ-retroviruses that were being used at that time. LVs can introduce their genes into non-dividing cells, which is useful for gene delivery to post-mitotic cells, such as neurons, retina, hepatocytes, and relatively quiescent hematopoietic stem cells (HSCs), and enables shorter ex vivo culture time—γ-retroviral vectors require more prolonged culture with multiple cytokines to induce cell division. The native genome size of HIV-1 (~10 kb) is larger than that of the murine gamma-retroviruses (~8 kb) and thus LVs can carry somewhat larger transgene sequences. The ability to carry larger transgene sequences enables the inclusion of the complex β-globin gene cassettes identified as necessary for high-level, erythroid-specific expression (e.g., reverse orientation, LCR HS elements, introns). Moreover, the REV/RRE axis of HIV-1 affords better nuclear export of the intact vector genomes for more effective delivery of intact transgenes.

From the beginning, LVs were designed with an improved safety profile compared to earlier γ-retroviral vectors. LVs incorporate a “self-inactivating” (SIN) property, resulting from a 133 bp deletion encompassing TATA and transcription factor binding sites in the enhancer/promoter of the U3 region of the viral long-terminal repeat (LTR) [46]. The partial deletions of the LTR eliminate viral promoter/enhancer activities and minimize the transactivation of neighboring promoters at sites of integration. Thus, transcription of a provirus is not driven by endogenous LTR promoters, unlike previous retroviral vectors, but is controlled by an internal exogeneous promoter, such as Cytomegalovirus (CMV) or phosphoglycerate kinase (PGK) promoters, or endogenous β-globin promoter for erythroid-specific expression.

**Figure 3:**
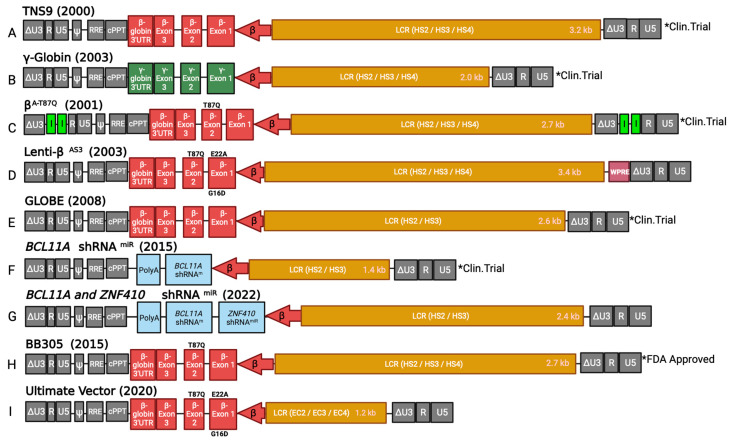
Proviral schematic of lentiviral vectors for β-hemoglobinopathies. All lentiviral vectors are self-inactivating (SIN) lentiviral vectors. β-globin vectors contain a wildtype β-globin gene, β-globin promoter, and Locus Control Region (LCR) made up of Dnase I hypersensitive sites HS2, HS3, and HS4, unless otherwise noted. (**A**) TNS9 vector, the first lentiviral β-globin lentiviral vector, containing 3.2 kb LCR. (**B**) γ-globin vector containing the γ-globin gene, and a 2.0 kb LCR. (**C**) β^T87Q^ vector β-globin vector containing a modified β-globin gene with an anti-sickling T87Q amino acid substitution, insulator elements, and a 2.7 kb LCR. (**D**) Lenti-β^AS3^ vector containing three anti-sickling amino acid changes (T87Q, E22A, and G16D), a 3.4 kb LCR and WPRE. (**E**) GLOBE vector β-globin vector with 2.6 kb LCR elements made of HS2 and HS3 elements only. (**F**) BCL11A shRNA^miR^ vector containing BCL11A shRNA^miR^, synthetic polyA, and 1.4 kb LCR of HS2 and HS3 elements only. (**G**) BCL11A and ZNF410 shRNA^miR^ vector containing BCL11A and ZNF410 shRNA^miRs^, synthetic polyA, and a 2.4 kb LCR constituted of HS2 and HS3 elements only. (**H**) BB305 vector β-globin vector containing the anti-sickling T87Q amino acid substitution, a 2.7 kb LCR region, and was approved by the U.S. Food and Drug Administration (FDA) in 2022. (**I**) Ultimate Vector (UV) retaining anti-sickling variants T87Q, E22A, and G16D, and a 1.2 kb LCR made up of core regions of HS2, HS3 and HS4, denoted EC2, EC3 and EC4, respectively. Definitions: ∆U3- U3: region with of the viral long-terminal repeat (LTR) with 133 bp deletion of viral enhancer; ψ: packaging signal; cPPT, central polypurine tract; RRE, rev-response element; polyA, polyadenylation signal; I, insulator; WPRE, woodchuck hepatitis virus posttranscriptional regulator element; shRNA^miR^: microRNA-adapted short hairpin RNA; *Clin. Trial: Clinical trials, currently or previously assessed in clinical trials. *FDA Approved: Completed clinical trials and has been approved for treatment.

In 2000, the first successful therapeutic use of β-globin LV was demonstrated by Sadelain and colleagues [47]. This β-globin LV, termed TNS9, contains a 3.2 kb LCR element (HS2, HS3, and HS4), has an intron 2 internal deletion and the β-globin gene is regulated by a 625 bp endogenous β-globin promoter (Figure 3A). TNS9 conferred high gene transfer efficiency into murine HSPCs and achieved long-term, elevated erythroid-specific expression when introduced into murine β-thalassemia marrow transplanted to irradiated mice. RBCs from the bone marrow of Hbb^th3/+^ β-thalassemia mice following transduction were characterized by improved erythrocyte morphology. The phenotypic rescue accomplished with the TNS9 β-globin LV had finally demonstrated the potential efficacy needed to treat β-hemoglobinopathies, such as β-thalassemia and sickle cell disease through gene therapy. Thus, extensive translational research involving high titer production of β-globin LVs with elevated erythroid-specific expression was further spurred. In 2012, this TNS9 LV was investigated in a clinical trial for severe β-thalassemia patients, the first trial in the United States for a severe inherited globin disorders (NCT01639690); interim results have been reported for four subjects in this study who achieved low level but persistent gene marking, with two having a reduced need for RBC transfusions [48]. Although not a fully comprehensive list, many of the clinically relevant β-globin LVs for sickle cell disease and β-thalassemia are described below.

## 5. Lentiviral Vectors for Sickle Cell Disease & β-Thalassemia

Sickle cell disease is caused by a point mutation that replaces a polar glutamic acid with a non-polar valine (βV6) on the surface of the β-globin chain of the hemoglobin tetramer (HbS, composed of 2 β^S^-globin and 2 α-globin chains). In the deoxygenated state, βV6 interacts with a natural hydrophobic domain in a β-globin chain (either wild type or mutated β-globin chain) of an adjacent hemoglobin molecule to initiate an aggregation process that leads to the polymerization of HbS tetramers into long fibers. It is important to recognize that attaining therapeutic benefits for sickle cell disease does not simply require the production of functional hemoglobin but also necessitates the reduction or inhibition of HbS polymerization, from which the vaso-occlusive crisis occurs.

Prior studies established that β^S^-globin initiates HbS polymerization by binding threonine 87 (T87) of the β-globin chain of an adjacent HbA. In parallel, studies revealed that γ- and δ-globin are excluded from HbS polymers [28]. The different amino acid composition found in both γ- and δ-globin chains, one of them being the polar and elongated glutamine 87 (Q87), was thought to be partly responsible for the anti-sickling effect. Indeed, this amino acid variation was demonstrated to prevent the formation of the nonpolar pocket that interacts the βV6, impeding HbS from binding and decreasing overall HbS polymerization and RBC sickling [28].

### 5.1. Anti-Sickling γ-Globin Lentiviral Vectors

Elevated levels of γ-globin production through hereditary persistence of fetal hemoglobin (HPFH) have been demonstrated to reduce clinical severity in patients with sickle cell disease [8,9]. Such clinical benefits come from the Q87 amino acid variation, not present in the β-globin chain, which impedes aggregation to HbS as described above. Therefore, treatments to induce HbF, such as hydroxyurea, have been of major interest and have ultimately shown to be successful in reducing sickle cell disease clinical adverse events [9,40,49]. HPHF is also clinically beneficial to patients with severe β-thalassemia by the provision of γ-globin chains to bind to unpaired α-globin chains and produce functional HbF [39,50,51]. An older broad study encompassing 272 patients with sickle cell disease examined the threshold of HbF induction for a major reduction of morbidity. While they suggest that there is no direct linear correlation between HbF levels and morbidity, they observed a reduction in major organ failure with a threshold of 10% of HbF, and a reduction of clinical events, including crisis or pulmonary events, in patients with >20% HbF [8].

To exogenously increase the expression of γ-globin for its anti-sickling activity, LVs were developed to carry the *HBG1* or *HBG2* gene in erythroid cells from transduced HSPCs. Persons and colleagues demonstrated high-level, therapeutic expression of a γ-globin LV in a Berkeley sickle cell disease mouse model; the vector bears a minimal β-globin promoter driving expression of γ-globin exons and selected parts of a 2.0 kb LCR with HS2, HS3, and HS4 regulatory elements (Figure 3B) [52]. Ultimately, they demonstrated in vivo correction of the sickle phenotype and reduction of splenic, renal, and hepatic sequelae in the sickle cell disease murine model, with approximately 95% F-cells with 40% induced HbF of total Hb [53]. Similarly, Malik and colleagues modified a previous β-globin LV to incorporate the γ-globin exons alongside the minimal β-globin promoter and the HS2, HS3, and HS4 elements of the β-globin LCR [54]. This γ-globin LV, denoted sG^b^G, demonstrated in vivo correction of the sickle phenotype and sickle cell-related sequelae in the Berkeley sickle cell disease mouse model treated with reduced intensity conditioning. Furthermore, by quantifying the HbF/F-cell of transduced mice through biotin surface labeling and intracellular HbF staining, they suggested that HbF/F-cell greater than one-third of the total hemoglobin in sickle RBC is sufficient to correct the sickle phenotype [54]. This vector is currently undergoing a Phase I/II clinical evaluation (see below) [55].

A markedly different LV, GGHI-mB-3D γ-globin LV, is being examined for potential treatments for sickle cell disease and β-thalassemia through HbF induction via the incorporation of HPHF elements. The newly optimized GGHI-mB-3D LV, based on a previous GGHI LV from the same group, is an β-globin LCR-free LV harboring the γ-globin gene flanked by novel regulatory elements from the γ- and α-globin loci. This vector yielded some improvement of the sickle cell disease and β-thalassemia phenotypes in patients’ differentiated HSPCs by observing a slight increase in HbF levels as well as improvements in erythroid maturation measured by the increase in the number of orthochromatic erythroblasts quantified by counts from Cytospin preparations of liquid cultures [56,57]. While this new vector may be produced to higher titer than vectors with a large LCR region, comparative studies using more standard LCR-bearing LVs would be useful, and in vivo assays should be done to determine their potential clinical efficacy.

### 5.2. Anti-Sickling β-Globin Variants

Concurrent with the development of anti-sickling γ-globin LV, investigators also developed LVs carrying designed anti-sickling β-globin variants. The first designed anti-sickling β-globin gene variant, β^A-T87Q^, carried a mutation in the β-globin gene replacing the 87 threonine (T87) within the hydrophobic pocket, with a protruding, polar glutamine, a variant referred to as T87Q. In 2001, Leboulch and collaborators developed the β^A-T87Q^ LV, containing a 2.7 kb LCR composing sections of HS2, HS3 and HS4, with a smaller intron 2 deletion than its predecessor TNS9 (Figure 3C) [58]. This anti-sickling β-globin LV improved sickle cell disease phenotype in a murine sickle cell disease model by significantly reducing sickled RBC and splenomegaly.

In 2003, the Townes laboratory developed a more potent anti-sickling β-globin variant, designated β^AS3^-globin, by substituting three amino acids within the β-globin gene. The first amino acid change was the β(T87Q) variant discussed above. The second modification, referred to as β(E22A), substituted glutamic acid 22 with alanine, to destabilize further the HbS interaction first described by his group in 1994 [59]. The β22 glutamic acid interacts with an imidazole group of the α20 histidine from an adjacent hemoglobin and plays a crucial role in HbS polymer stability. The substitution to a short and non-charged amino acid, such as alanine, present in γ- and δ-globin chains, disrupts this binding interaction. The third modification increases the affinity of β-globin subunits to the α-globin chain by replacing glycine 16 with aspartic acid (G16D), first observed in a natural non-pathogenic β-globin variant (hemoglobin J-Baltimore) [60]. Studies suggest that aspartic acid contains greater affinity to the α-chain and, therefore, increases the affinity to the α-globin polypeptide compared to the mutated β-chain during the formation of the hemoglobin tetramer [61].

A β^AS3^-globin LV, referred to as Lenti-β^AS3^, constructed in the Townes laboratory contained a 3.4 kb LCR (elements from HS2, HS3, and HS4) and β^AS3^-globin expression is driven by a 265 bp β-globin minimal promoter (Figure 3D). This vector further established the efficacy of β-globin variant LVs, as it corrected abnormal RBC morphology and ameliorated spleen, liver and kidney pathology in sickle cell disease mice [62]. Further analysis showed that 82% of the tetramers formed contained β^AS3^-globin subunits, rather than the HBB^S^ (β6V) subunit, consistent with the expected function of the G16D substitution to increase affinity for α-globin. A β^AS3^-globin LV (GLOBE-AS3) was produced using the shorter GLOBE LV backbone. GLOBE is a LV developed by Ferrari and colleagues for the treatment of β-thalassemia carrying a wild type β-globin gene and a 2.6 kb LCR with HS2 and HS3 elements and has recently completed clinical trials studies (Figure 3E) [63,64,65].

### 5.3. Induction of HbF for Anti-Sickling Activity

Silencing of *BCL11A,* one of the main γ-globin repressors, allows for HbF production, which in turn decreases the polymerization of HbS [66]. Studies showed that knocking out *BCL11A* in mouse models not only corrected sickle cell disease phenotypes but also mitigated disease symptoms [67].

MicroRNA-adapted short hairpin RNAs (shRNA^mir^) have been incorporated into LVs to promote the silencing of the regulatory gene *BCL11A* for therapeutic benefits in sickle cell disease. These shRNA^mirs^ are modified shRNAs with flanking regions designed from miRNA scaffolds to allow the use of a polymerase II promoter that enables cell and lineage-specific targeting with low passenger strand activity [68]. The *BCL11A* shRNA^mir^ was first shown to be advantageous in reactivating γ-globin expression when transduced into primary HSPCs and differentiated into erythroid cells (Figure 2B). Not only were expression levels of γ-globin comparable to RNA polymerase III promoter-based systems, but they also produced minimal toxicity to the engrafting HSPCs and other hematopoietic lineages due to the absence of ectopic pan-hematopoietic expression [68]. The *BCL11A* shRNA^mir^ LV, carrying a short 1.4 kb LCR with HS2 and HS3 elements, was studied in vivo using the Berkeley-sickle cell disease mouse model for gene therapy (Figure 3F). It provided therapeutic benefits in alleviating sickle cell disease symptoms and demonstrated success for erythroid-specific BCL11A knockdown in preventing toxic effects in HSCs during engraftment [66]. Currently, this gene therapy strategy has shown clinical benefits in clinical trials (see below) [69]. Another target for γ-globin induction involves knocking down *ZNF410* with shRNA^miR^ technology. LVs containing both *ZNF410* and *BCL11A* shRNA^miRs^ led to greater γ-globin induction than *BCL11A* shRNA^miR^ vectors (Figure 3G). Sickle cell disease patient HSPCs that have been treated with a vector containing both a *ZNF410* and a *BCL11A* shRNA^miRs^ and differentiated into erythrocytes led to an increase of 10% HbF and a significant decrease of sickled cells in comparison to *BCL11A* shRNA^miR^ only vector treated cells [70]. Hence, the *ZNF410* and *BCL11A* shRNA^miR^ vector shows a greater potential and application in both sickle cell disease and β-thalassemia therapy.

## 6. Sickle Cell Disease and β-Thalassemia Gene Therapy Trials

Results in preclinical studies from the past two decades demonstrated strong disease amelioration in sickle cell disease and β-thalassemia mouse models, which prompted investigators internationally to start clinical trials using a β-globin LV for patients with severe cases of both conditions. Trials for gene therapy of sickle cell disease and β-thalassemia began in 2007 in Europe and in 2013 in the United States (Table 1).

The first β-thalassemia patient was treated with the β^A-T87Q^ lentivector, or LentiGlobin HPV569, and showed significant improvement in their anemia and became transfusion independent for at least several years [71]. After gene therapy, the patient had approximately 1/3 of the hemoglobin in RBC containing the anti-sickling β^T87Q^-globin chain, the amino acid substitution acting as a marker. However, this level of anti-sickling β-globin was largely due to a clonal expansion of an erythroid progenitor cell containing the LV. In one HSPC, the vector integrated within an intron of the *HMG2A* gene and dysregulated the gene’s expression by interfering with RNA splicing and eliminating downstream microRNA binding sites involved in regulating HMG2A expression through RNA stability [71]. Fortunately, this clone became less prominent over time and did not progress to a leukoproliferative state. However, the total level of gene-corrected stem cells was relatively low, besides the HMG2A adjacent integrant clone.

LentiGlobin HPV569 was further modified through the removal of the insulator elements to improve titer and gene transfer [72]. The resulting vector, referred to as LentiGlobin BB305, was used in clinical trials for β-thalassemia sponsored by bluebird bio ®, yielding the first gene therapeutic for hemoglobinopathies in the United States (Figure 3H) [72,73]. In trials performed in the United States and France, 22 thalassemia patients were treated [74]. Twelve of thirteen with the slightly milder β^0/^βE genotype produced sufficient transgenic β-globin to become transfusion independent; in line with the more severe β^0/^β^0^ genotype, transfusion requirements were significantly reduced and three were reported to have become transfusion independent. Based on these and additional positive results, bluebird bio’s gene therapy was approved by the European Medicines Agency in June 2019 and by the FDA in October 2022.

Ferrari and colleagues at the San Raffaele Telethon Institute for Gene Therapy have also reported a clinical trial using their GLOBE LV, a smaller size than the vector used in the bluebird trials, for gene therapy of β-thalassemia (Figure 3E) [63]. They achieved reduced transfusion requirements in three adult patients, and three of four pediatric patients with blood transfusion-dependent β-thalassemia became transfusion independent [75].

The first report of a single successful case of gene therapy for sickle cell disease boldly demonstrated the potential efficacy of this approach [76]. A thirteen-year-old boy with severe sickle cell disease on chronic RBC transfusion therapy underwent gene therapy using the β^A-T87Q^ LV from bluebird bio to transduce bone marrow stem cells which were reinfused after cytoablative marrow conditioning with busulfan chemotherapy. He achieved sufficient levels of gene-modified stem cells and with RBC expressing ~50% of β^T87Q^-globin to appreciate marked clinical improvement.

Other adult patients treated in an initial United States trial using the same vector did not show the same relatively high level of engraftment of gene-modified stem cells, expressed only low levels of HBB^T87Q^, and did not realize clinical benefit [77]. However, there were successive modifications to the clinical protocol and cell processing methods that are leading to consistent clinical successes [78]. Peripheral blood stem cells (PBSC) were mobilized by the CXCR4 inhibitor plerixafor which provides more cells than a bone marrow harvest. Lead-in RBC transfusions were given for a few months before mobilization and leukapheresis to “calm” the HSPCs and marrow microenvironment [78]. An improved cell transduction process was used incorporating transduction enhancer compounds, such as poloxamer, PGE2, protamine sulfate or other agents, to improve gene transfer by the large β-globin LV.

More recently, these investigators reported results in 35 sickle cell disease patients by the refined method [78]. All 25 evaluable patients had a complete cessation of vaso-occlusive crises, which had averaged 3.5 episodes per year before treatment. On average, more than 40% of the hemoglobin in RBC was the T87Q anti-sickling globin from the vector. Bluebird bio has announced that they plan to file for FDA licensure of this gene therapy for sickle cell disease.

Other academic-based clinical trials are using LVs to evaluate other anti-sickling strategies. Malik and colleagues developed a LV with the exons from γ-globin and the transcriptional control elements for β-globin [79,80]. They recently reported that three treated patients had relatively low levels of gene-corrected cells (e.g., vector copies/cell average of 0.15–0.2) but have had improvements in clinical symptoms. Our group at UCLA is using the β^AS3^-globin gene incorporated into the GLOBE LV backbone [62,81]. One patient was treated in 2015, using similar clinical and cell processing methods as in the early bluebird studies and only had a low level of gene-corrected cells engrafted with no clinical benefit. A modified version of the protocol is open and recently has treated more subjects.

Esrick, Williams and colleagues used their LV-producing shRNA^mir^ to the mRNA of BCL11A LV in a clinical trial for sickle cell disease (Figure 3F) [66]. They have reported eight patients treated by autologous HSCT with this vector, with three patients with a 9–18-month follow-up after gene therapy. They reached 24–44% HbF with marked decreases in clinical symptoms and more subjects have been enrolled [69]. A multi-center Phase II trial of the shRNA^mir^ to BCL11A led by these investigators is underway.

## 7. Evolving Technologies to Further Improve Gene Therapy for β-Hemoglobinopathies

Challenges to optimize vector manufacturing processes and HSPC transduction efficiency remain important obstacles limiting the potential clinical success of gene therapy for hemoglobulin gene therapies. Despite the ability of LVs to transduce dividing and non-dividing cells, the transduction of human HSPCs may be further improved to reach more consistent therapeutic efficiency. One factor is the inherent relative resistance of human HSPCs to lentivirus infections. While the mechanism(s) underlying HSPC resistance are not well understood, small molecules such as Cyclosporin H, Rapamycin, and poloxamer 338, have improved transduction efficiency.

Another principal factor that reduces viral titer is the large size of the β-globin genome arising from the genomic sequences, including, exons, introns, 3′UTR, and LCR, necessary for high-level erythroid-specific gene expression. In addition to their large size, some of these genomic regions can result in problematic sequences, such as cryptic polyadenylation signals and splicing sites, which become operant when transcribed in reverse orientation, which is required to retain the β-globin introns during LV packaging. In 2020, our laboratory set out to determine whether the proviral size or specific β-globin sequences were the main determinants for low titer and sub-optimal gene transfer observed in similar β-globin LVs. Our data reaffirmed that genome length, rather than the presence of any specific adverse sequences, was the major obstacle impeding vector functions [82]. As such, Morgan and team developed a β-globin LV, termed Ultimate Vector (UV), containing a 1.2 kb LCR with “Encode cores” (EC) of HS2, HS3, and HS4 (referred to as EC2, EC3, and EC4, respectively), which is about 3.5 kb smaller than parental β-globin vectors (Figure 3I). This UV LV is therapeutically promising because of its smaller proviral length, yielding a high titer and improved gene transduction of CD34+ cells, although with reduced gene expression levels. While the level of expression per vector copy number is lower, the ability to transduce more HSPCs yields a similar net β-globin gene expression level. Furthermore, the higher titer and more efficient transduction of CD34+ HSPC by UV would lead to many more patient doses of vector per production lot, lowering the costs for that component of the gene therapy product. Additionally, an LV newly developed in our group in collaboration with the laboratory of David Williams incorporates the two γ-globin induction strategies into the UV backbone (β^AS3^-globin with shRNA^mir^ to both BCL11A and ZNF410) thereby leading to a highly optimized vector with high titer, efficient HSPC transduction, and high levels of the two types of anti-sickling globin (via the β^AS3^-globin transgene and the induction of endogenous HbF strategies) [70,83].

## 8. Use of Chromatin Insulators in LV

Overall, there have been no reports of vector-related leukemia to date in the several hundred individuals who have undergone gene therapy with LVs, for conditions other than sickle cell disease (with the single exception of myelodysplastic syndrome observed in a patient with cerebral adrenoleukodystrophy treated with a lentiviral vector that used a potentially transforming retroviral long terminal repeat enhancer to drive expression), including at least 41 β-thalassemia patients who received the identical vector, LentiGlobin BB305. Chromatin insulators, such as the chicken hypersensitive site 4 (cHS4), were included in some clinical LVs (e.g., LentiGlobin HPV569, the prior version of the BB305 vector, with two copies of cHS4 in each LTR), to potentially provide additional safety by inhibiting trans-activation of cellular genes by enhancers of the integrated vector. However, studies suggest that cHS4 insulating function is variable, dependent on the chromatin site, and prone to rearrangement which further weakens its function as a protective element [71,84,85]. It also contains a cryptic splice acceptor site, which has been associated with sub-clinical genotoxicity and clonal expansion in two studies (β-thalassemia and X-linked SCID) [71,86].

Our group has previously investigated the integration of a synthetic insulator element, FB, that has the core CTCF binding sites from the cHS4 element and the human TCR γδ gene’ the FB insulator showed a 12-fold enrichment of CTCF binding to LTR of LVs in vitro [81]. This FB element has therefore remained in a LV, Lenti/G-βAS3-FB LV, currently in a clinical trial for sickle cell disease at UCLA. Romero et al. have also evaluated the integration of the human ankyrin 1 promoter insulator in a β-globin LVs and found that integration of such insulator increased long-term β-globin gene expression in murine HSPCs, as demonstrated in secondary transplants [87]. Because no significant benefits have been seen with insulators, their removal from β-globin SIN LVs is considered more advantageous as it also generates shorter vectors resulting in optimized titer production and gene transfer efficiency.

## 9. Gene Editing as an Alternative to LV

In the past decade, direct gene editing for genetically modifying cells has emerged as an alternative modality to gene addition, such as with LV. In theory, site-directed editing may reduce the risks of genotoxicity from semi-randomly integration viral vectors. Site-specific endonucleases, such as Zinc Finger Nucleases, TALENs and especially the Clustered Regularly Interspaced Short Palindromic Repeats (CRISPR) and CRISPR-associated (Cas) system have been extensively studied and optimized for its usage in gene therapies, especially for sickle cell disease and β-thalassemia [88]. By introducing a double-stranded DNA break (DSB) at the genomic site to be edited, cellular DNA repair pathways are induced which can either (1) re-anneal the cut strands through a somewhat error-prone mechanism, non-homologous end joining (NHEJ) often introducing insertions or deletions (indels) at the site, or (2) resolve the DSB by a more precise mechanism that uses a provided nucleic acid “donor” to provide the corrective sequences which are pasted into the DSB site by homology-directed repair (HDR). For HSPCs, the cells are typically electroporated to introduce the editing reagents (e.g., recombinant Cas9 protein or mRNA and a single guide RNA {sgRNA}, plus the homologous donor as either a deoxyoligonucleotide or carried by a non-integrating viral vector, such as AAV6. Iterative improvements in the quality of the editing reagents and the cell manipulation methods have led to very high-efficiency gene editing, especially for gene disruption approaches.

Recent clinical trials present encouraging data from utilizing CRISPR-Cas9 to disrupt the erythroid enhancer of *BCL11A* for the induction of HbF (NCT03655678, NCT03745287). Highly effective results have been reported, leading to sufficient HbF expression to essentially eliminate subsequent sickle crises in the majority of subjects. Other trials will attempt to correct the canonical mutation in *HBB* that causes sickle cell disease, using HDR and homologous donors to provide the corrective base. A long-term follow-up clinical trial is underway. However, CRISPR-Cas9 induced DSBs bring their own risks and limitations upon repair, displaying adverse survival and genotoxic effects on HSPCs. These include insertions/deletions of varying length, chromosomal translocation, and chromothripsis [89,90,91,92].

A more recent genome editing approach, developed by Liu’s group, is known as base editing and employs the Cas9/sgRNA system to bring to the editing site deaminase enzymes to modify a single base (e.g., convert adenine to guanine or cytosine to thymine) [89]. The main benefit of this novel gene therapy approach is that it is efficient and precise, and only uses a single-strand nick, which may be safer than inducing DSB. Base editors are already making their way to the clinical trials for both sickle cell disease and β-thalassemia (NCT05456880). Even newer versions of editors include Prime Editing, which may insert a few specific base pairs at an editing site, and CRISPR/Transposases and Large Serine Recombinases, which may be able to insert longer sequences (e.g., cDNA size) without causing DSB or needing HDR to occur.

## 10. Bone Marrow Conditioning to Facilitate Engraftment of Gene-Modified HSC

To facilitate the engraftment, chemotherapy agents are used to deplete the bone marrow HSPCs which creates space for the infusion of the newly modified cells. This process, known as myeloablation conditioning, employs alkylating agents (such as busulfan) that replace alkyl groups found in DNA with oxygen atoms which cause DNA crosslinks, abnormal DNA base pairing or strand breaks which prevents HSPCs from undergoing replication and leads to their elimination; busulfan is overall less toxic than total body irradiation [93,94]. While this conditioning has been effective in patients undergoing HSCT-GT, chemotherapy can lead to infertility, infections and off-target genotoxicity potentially leading to secondary malignancy. The latter risk is compounded by disease settings seen in sickle cell disease where patients have chronic inflammation, hypoxemia, and expended hematopoiesis and as a result contain increased mutations [95]. Indeed, a California study has reported a 72% higher risk of hematologic malignancies in patients with sickle cell disease [96]. A recent long-term follow-up for gene therapy for sickle cell disease using BB305 vector (NTC02140554) diagnosed two of the participants with myelodysplastic syndrome (MDS) which eventually developed into acute myeloid leukemia (AML). All analyses supported busulfan conditioning to be the cause in the first patient, rather than mediated insertional oncogenesis, as multiple independent assays demonstrated absence of vector integration in CD34^+^ blast, along with complex cytogenetic abnormalities and driver gene mutations [97]. In the second patient, there was a lentiviral vector integrant in the leukemic clone, but it was near a gene thought to have no oncogenic potential. Instead, the favored hypothesis is that there is accelerated acquisition of leukemogenic mutations in the marrow stem cells of sickle cell disease patients. Poor engraftment from allogeneic donors or of gene-modified autologous stem cells allows the pre-existing clones bearing proliferation-promoting mutations to expand.

Genotoxic conditioning regimens using chemotherapy agents, however, are still required for almost all HSCT-GT to attain significant levels of HSC engraftment, which adds tangible risks to the therapy (Fanconi’s Anemia presents an exception because the severe compromise of the HSC compartment by the inherent DNA repair defect allows engraftment of gene-modified HSC with minimal or no conditioning). Conditioning regimens and their intensity are generally adjusted for the disease type (lower intensity can be used when there is a selective advantage to the key blood cell lineage) and dose adjusted based on measurements of blood levels and clearance for each patient to minimize toxicity while still supporting therapeutic levels of engraftment. To eliminate the risk from chemotherapy conditioning, several groups have been examining non-genotoxic conditioning regimens using monoclonal antibodies targeting specific HSPC markers. One of these strategies considered for gene therapy conditioning employs a monoclonal antibody targeting the cell-surface receptor c-kit (CD117), known as Briquilimab (also known as JSP191). Briquilimab is currently being evaluated as nonmyeloablative HSCT for sickle cell disease and β-thalassemia in a phase I/II clinical trial (NCT05357482) and has had preliminary successful and safe outcomes when utilized for the conditioning regimen of HSCT for AML and MDS [98]. Another group is currently investigating a CD117 antibody-drug conjugate (ADC) for HSCT-GT in non-human primates (NHPs), prior to reinfusion of NHP CD34+ previously transduced with β-globin LV [99]. Preliminary data showed that one dose of the ADC resulted in a fully myeloablative effect with greater than 99% HSPC depletion, and was well-tolerated with no adverse effects, unlike NHPs treated with high dose busulfan. An additional nongenotoxic conditioning approach to aid engraftment of ex vivo transduced HSPCs involves the transient modification of HSPC utilizing mRNA constructs encoding homing receptors, like CXCR4, or survival factors such as BCL2 [100].

## 11. Conclusions, Looking Forward

Autologous HSCT-GT using LV (and more recently gene editing) has demonstrated curative effects for sickle cell disease and β-thalassemia. Such therapy circumvents the biggest limitation and risks of allogeneic HSCT, which arise from the necessity for an immunologically matched donor and risks from immunological rejection or graft versus host disease. HSCT-GT has the potential to be available to essentially all patients, serving as their own perfectly matched donor.

This autologous ex-vivo gene therapy, however, may remain an elite treatment, regardless of its efficacious clinical trial results. The high price of the procedure mostly results from the manufacturing and clinical infrastructure needed, and the extensive medical and scientific training required—which, overall, makes this ex-vivo autologous gene therapy inaccessible in developed as well as developing countries in which most sickle cell disease and β-thalassemia patients reside. The biggest challenge for scientists is to find ways to make this curative approach an accessible and affordable one. As of now, one ambitious, but foreseeable, goal is to evolve these gene therapy techniques to an in vivo delivery mode, where systemic injection of a vector or editing reagents effectively corrects the *HBB* gene in HSPCs in situ. This approach would not only simplify the current methodology that necessitates marrow cytoablation and all the clinical support required for HSCT but should also significantly decrease the cost. Delivery of these reagents diffusely to the marrow throughout the skeleton remains a major challenge, but efforts are underway (e.g., using Ad35/5 vectors) [101].

Decades of unwavering and collaborative studies around the world in globin research have not only elucidated some of the most exciting molecular discoveries but have also led to pioneering work on the development and usage of viruses as biological tools, which are now widely employed for experimental biological applications. Such scientific innovation, drive, and dedication is still indispensable to transform these revolutionary therapies into accessible ones for all patients, and into what can and should be one of the most exciting and necessary medical advances of the 21st century.

## Figures and Tables

**Table 1:** Sickle Cell Disease and β-thalassemia Clinical Trials.

	Sponsor	Country	Year	Disease	Lentiviral Vector Name	Participant	Status	Clinical Trial Identifier
Beta Globin Gene Insertion	Genetix Pharmaceuticals (later, renamed bluebird bio)	France	2006	β-thalassemia Major	LentiGlobin HPV569 Lentiviral β-A-T87Q Globin Vector	2	Completed, Phase 1 and 2	n/a
	Memorial Sloan Kettering Cancer Center	USA	2012	β-thalassemia Major	TNS9.3.55 Lentiviral encoding WT β-globin gene (Figure 3A)	4	Active, Phase 1	NCT01639690
	bluebird bio	France	2013	β-thalassemia Major Sickle Cell Disease	LentiGlobin BB305 Lentiviral β-A-T87Q Globin Vector (Figure 3H)	7	Completed, Phase 1 and 2	NCT02151526
	bluebird bio	USA	2014	Sickle Cell Disease	LentiGlobin BB305 Lentiviral β-A-T87Q Globin Vector (Figure 3H)	50	Active, Phase 1 and 2	NCT02140554
	bluebird bio	USA Australia Thailand	2013	β-thalassemia Major	LentiGlobin BB305 Lentiviral β-A-T87Q Globin Vector (Figure 3H)	19	Completed, Phase 1 and 2	NCT01745120
	bluebird bio	USA France Germany Italy Thailand United Kingdom	2016	β-thalassemia–transfusion dependent excluding β-thalassemia Major	LentiGlobin BB305 Lentiviral β-A-T87Q Globin Vector (Figure 3H)	23	Completed, Phase 3	NCT02906202
	bluebird bio	USA France Germany Greece Italy UK	2017	β-thalassemia–Transfusion Dependent	LentiGlobin BB305 Lentiviral β-A-T87Q Globin Vector (Figure 3H)	18	Active, Phase 3	NCT03207009
	bluebird bio	USA	2020	Sickle Cell Disease	LentiGlobin BB305 Lentiviral β-A-T87Q Globin Vector (Figure 3H)	35	Recruiting, Phase 3	NCT04293185
	IRCCS San Raffaele Orchard Therapeutics	Italy	2015	β-thalassemia	GLOBE Lentivirus Vector Lentiviral vector with wild type β-globin gene (Figure 3E)	10	Completed, Phase 1 and 2	NCT02453477
	Donald B. Kohn, M.D. California Institute for Regenerative Medicine	USA	2014	Sickle Cell Disease	Lenti/G-βAS3-FB Lentiviral Vector Lentiviral containing anti-sickle β-AS3 (not shown)	6	Recruiting, Phase 1 and 2	NCT02247843
	Children’s Hospital Medical Center, Cincinnati	USA	2014	Sickle Cell Disease	Gamma Globin Lentivirus Vector Lentiviral vector with fetal γ-globin gene (Figure 3B)	7	Active, Phase 1 and 2	NCT02186418
Fetal Induction	David Williams, M.D.	USA	2017 2022	Sickle Cell Disease	short-hairpin RNA targeting BCL11A Lentiviral Vector Lentiviral vector with short-hairpin BCL11A RNA (Figure 3F)	10 25	Active, Phase 1 Recruiting, Phase 2	NCT03282656 NCT05353647
